# Epilepsy professionals' views on sudden unexpected death in epilepsy counselling: A tale of two countries

**DOI:** 10.1111/ene.16375

**Published:** 2024-06-04

**Authors:** Lance Watkins, Oliver Henning, Paul Bassett, Samantha Ashby, Samuel Tromans, Rohit Shankar

**Affiliations:** ^1^ University of South Wales Pontypridd UK; ^2^ Swansea Bay University Health Board Port Talbot UK; ^3^ Cornwall Intellectual Disability Equitable Research (CIDER) University of Plymouth Peninsula School of Medicine Truro UK; ^4^ National Epilepsy Center Oslo University Hospital Oslo Norway; ^5^ Statsconsultancy Ltd Bucks UK; ^6^ SUDEP Action Wantage UK; ^7^ SAPPHIRE Group, Department of Population Health Sciences University of Leicester Leicester UK; ^8^ Adult Learning Disability Service Leicestershire Partnership NHS Trust Leicester UK; ^9^ Cornwall Intellectual Disability Equitable Research (CIDER) Cornwall Partnership NHS Foundation Trust Truro UK

**Keywords:** education and training, epilepsy harm, epilepsy mortality, epilepsy risk, seizure‐related death

## Abstract

**Background and purpose:**

Sudden unexpected death in epilepsy (SUDEP) is a leading cause of epilepsy mortality. All international guidance strongly advocates for clinicians working with people with epilepsy (PWE) to discuss SUDEP. Clinician views working with PWE in the UK and Norway on SUDEP counselling are compared.

**Methods:**

A cross‐sectional online mixed methodology survey of 17 Likert and free‐text response questions using validated themes was circulated via International League against Epilepsy/Epilepsy Specialist Nurses Association in the UK and International League against Epilepsy/Epilepsinet in Norway using a non‐discriminatory exponential snowballing technique leading to non‐probability sampling. Quantitative data were analysed using descriptive statistics and Mann–Whitney, Kruskal–Wallis, chi‐squared and Fisher's exact tests. Significance was accepted at *p* < 0.05. Thematic analysis was conducted on free‐text responses.

**Results:**

Of 309 (UK 197, Norway 112) responses, UK clinicians were more likely to have experienced an SUDEP (*p* < 0.001), put greater importance on SUDEP communication (*p* < 0.001), discuss SUDEP with all PWE particularly new patients (*p* < 0.001), have access and refer to bereavement support (*p* < 0.001) and were less likely to never discuss SUDEP (*p* < 0.001). Significant differences existed between both countries’ neurologists and nurses in SUDEP counselling with UK clinicians generally being more supportive. UK responders were more likely to be able to identify bereavement support (*p* < 0.001). Thematic analysis highlighted four shared themes and two specific to Norwegians.

**Discussion:**

Despite all international guidelines stating the need/importance to discuss SUDEP with all PWE there remain hesitation, avoidance and subjectivity in clinicians having SUDEP‐related conversations, more so in Norway than the UK. Training and education are required to improve communication, engagement and decision making.

## INTRODUCTION

Sudden unexpected death in epilepsy (SUDEP) is defined as sudden, unexpected, witnessed or unwitnessed, nontraumatic and non‐drowning death in an individual with epilepsy, with or without evidence for a seizure and excluding documented status epilepticus where postmortem examination does not reveal a cause for death [1, 2, 3, 4, 5, 6, 7, 8].

Although there are currently no established evidence‐based prevention strategies, in the past couple of decades awareness of SUDEP has grown [9, 10]. PWE, their families and caregivers want more information about SUDEP from healthcare professionals [7]. The existing evidence shows a discrepancy between what clinicians identify as important and for whom, and what PWE expect [11]. National and international guidelines outline that SUDEP should be discussed at the earliest appropriate time with every person with epilepsy [12, 13]. Consensus on good clinical practice indicates that PWE should be counselled on SUDEP risk at the first appointment and ideally at regular intervals [14, 15].

A recent review of healthcare professionals' views on SUDEP counselling evaluated the content of 16 studies [11]. From this, a range of essential and relevant question themes were identified. The question themes cover demographics, clinical experience and SUDEP‐specific domains. The findings provide an evidence‐based standardized approach to surveying healthcare professionals on SUDEP counselling that can be used for comparison between settings and over time [11].

In the UK (population 67.3 million), there are 1200 epilepsy‐related deaths a year of which approximately 60% are thought to have been avoidable [5, 9]. SUDEP accounts for approximately 500 of these deaths a year [5]. National guidance in the UK since 2004 has enumerated the need for a compulsory clinical conversation with people on epilepsy risks, particularly SUDEP [16]. Studies to date indicate that only a third of clinicians in the UK discussed SUDEP with PWE [16].

In Norway (population 5.4 million), it is estimated that there are around 130 epilepsy‐related deaths each year of which SUDEP accounts for at least 30 [17]. In Norway, over 90% of PWE surveyed wanted information on epilepsy‐related risks particularly SUDEP but less than a third (30%) told of receiving it [18].

UK and Norway, although having differences in population and health systems, are comparable across diverse socio‐demographic and health delivery outcomes [19]. There has been no re‐evaluation of healthcare professionals' attitudes towards SUDEP counselling in this decade in either country, especially given the significant raised profile of SUDEP communication due to publication of major influential international guidance [12, 13, 20, 21, 22]. Further, the impact of the pandemic on SUDEP counselling has not been explored in either country.

### Aim

The aim was to gather the views on SUDEP counselling of healthcare professionals who are actively involved in epilepsy care in the UK and Norway and draw a comparison between the two countries.

## METHODS

The STROBE guidance for cross‐sectional studies was followed and used to report this study (Data S1).

### Survey development

The survey questions (Data S2) were developed collaboratively by the authors using validated themes [11]. The survey had 17 questions (12 Likert style or single choice, five open response/comments).

### Ethics

Ethics was confirmed from the research ethics committee in Oslo University Hospital HF, application number 607647 (Data S3). In the UK the survey was reviewed by professional bodies, that is, the International League against Epilepsy (British branch) and the Epilepsy Specialist Nurses Association before dissemination to their members. All participants were advised at the start of the study that participation was voluntary and informed consent would be presumed if the survey was submitted. If they chose to participate, data would be pooled, anonymized and analysed. No participant identifier data were collected. Further, it was to a professional participant group where consent was implicit by participation.

### Participants and recruitment

A non‐discriminatory exponential snowballing technique leading to non‐probability sampling was used to disseminate the survey in electronic form.

### United Kingdom

The survey was distributed via the International League against Epilepsy British chapter and Epilepsy Specialist Nurses Association. The survey was open from 6 February 2023 to 2 April 2023.

### Norway

The survey was distributed via International League against Epilepsy Norway chapter, the Epilepsy Nurses National Network and the National Interdisciplinary Epilepsy Network (Epilepsinet). The survey was open from 12 April 2023 to 20 June 2023.

### Statistical analysis

The UK respondents were divided into four main categories (by professional role), and comparisons were undertaken between the four groups. For Norway, there were only sufficient numbers in two groups; thus inter‐group comparisons were focused on these two groups. Categorical variables with a natural ordering between groups were compared using the Kruskal–Wallis test. Categorical variables with no ordering to the categories were compared using the chi‐squared or Fisher's exact test. UK and Norway comparison analyses were performed for all respondents, and separately for neurologists and for nurses. Significance was accepted at *p* < 0.05.

### Analysis of free‐text responses

The five qualitative questions sought to explore views, expectations and concerns of the respondents relating to SUDEP communication through free‐text responses. Analysis was conducted collaboratively between three co‐authors. A descriptive thematic approach was chosen to enable respondent views to be presented in a way which is applicable to everyday healthcare practice and suitable to mixed methodology studies [23, 24].

## RESULTS

The survey received 309 (UK 197, Norway 112) responses from healthcare professionals working with PWE.

### United Kingdom

The 196 respondents were largely made up of neurologists (38%) (general/epileptologist/paediatric) and epilepsy nurses (38%). Other specialism areas represented were psychiatrists (7%) and general paediatricians (7%). Twenty‐eight per cent had less than 5 years' clinical experience in epilepsy, 31% had over 15 years' experience. Forty‐five per cent worked with PWE more than 75% of the time, whilst 19% of respondents worked less than a quarter of their time in epilepsy (Table 1).

**TABLE 1:** Background demographics and epilepsy factors, raw data.

Question	*n*	Category	*n* (%)	*n*	Category	*n* (%)
UK				Norway		
Job title	196	Neurologist – general	24 (12%)	112	Neurologist – general	33 (29%)
		Neurologist – epileptologist	23 (12%)		Neurologist – epileptologist	22 (20%)
		Epilepsy nurse specialist	74 (38%)		Epilepsy nurse specialist	21 (19%)
		Nurse – other	6 (3%)		Nurse – other	16 (14%)
		Psychiatrist – neuropsych.	1 (1%)		Psychiatrist – neuropsych.	1 (1%)
		Psychiatrist – int. disabilities	13 (7%)		Psychiatrist – int. disabilities	0 (0%)
		Psychiatrist – other	0 (0%)		Psychiatrist – other	0 (0%)
		General paediatrician	14 (7%)		General paediatrician	2 (2%)
		Paediatric neurologist	28 (14%)		Paediatric neurologist	0 (0%)
		Other	13 (7%)		Other	17 (15%)
Experience in epilepsy	195	0–5 years	54 (28%)	110	0–5 years	19 (17%)
		5–10 years	44 (23%)		5–10 years	23 (21%)
		10–15 years	36 (18%)		10–15 years	19 (17%)
		>15 years	61 (31%)		>15 years	49 (45%)
Percentage work epilepsy specific	196	<25%	37 (19%)	112	<25%	33 (29%)
		25%–50%	39 (20%)		25%–50%	21 (19%)
		50%–75%	32 (16%)		50%–75%	17 (15%)
		>75%	88 (45%)		>75%	41 (37%)
How often discuss SUDEP^a^	193	All patients	39 (20%)	112	All patients	2 (2%)
		Only new patients	100 (52%)		Only new patients	12 (11%)
		If change in risk	140 (73%)		If change in risk	44 (39%)
		Patients who ask	98 (51%)		Patients who ask	46 (41%)
		When remember to	5 (3%)		When remember to	7 (6%)
		Rarely or never	3 (2%)		Rarely or never	29 (26%)
Percentage time discussed SUDEP	178	<25%	162 (91%)	102	<25%	91 (89%)
		25%–50%	15 (8%)		25%–50%	11 (11%)
		>50%	1 (1%)		>50%	0 (0%)

*Note*: UK (*n* = 197) and Norway (*n* = 112) respondents.

Abbreviation: SUDEP, sudden unexpected death in epilepsy.

^a^ Respondents could answer in more than one category. Percentage values may not add up to 100%.

#### SUDEP discussion

**FIGURE 1:**
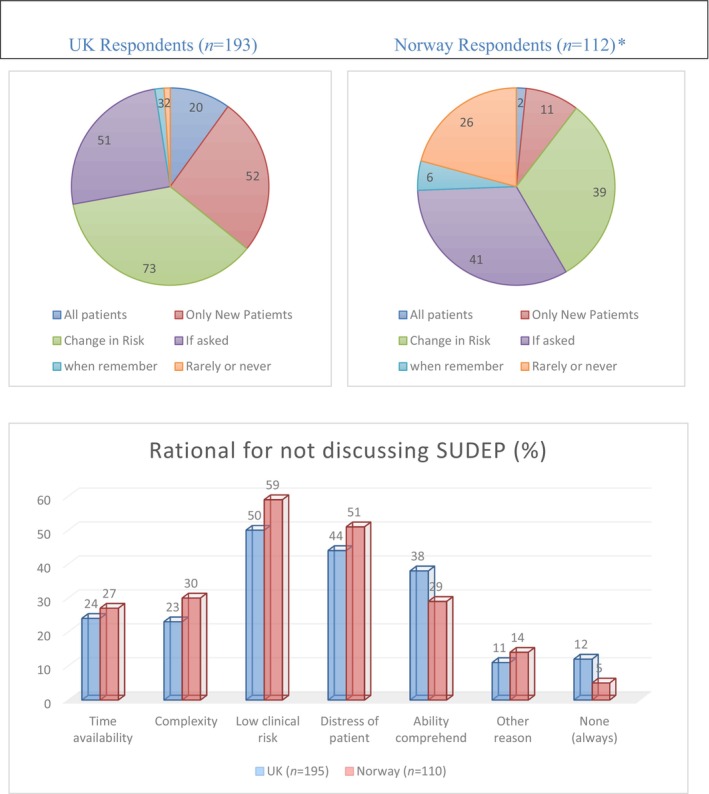
Comparison between UK and Norway respondents: SUDEP discussion. *Significant difference between UK and Norway responses across four categories (*p* < 0.001).

Of 193 respondents one‐fifth discussed SUDEP with ‘all patients’, 52% only discussing with ‘new patients’; 73% discussed SUDEP if they identified a change in ‘risk’ (clinician defined), and 2% rarely or never discussed SUDEP with any patient at any time. The majority (91%) indicated that SUDEP discussion was done by them in 25% or less of the time available for a routine patient consultation (Figure 1).

#### SUDEP awareness

Fifty‐six per cent of 193 respondents had experience of a SUDEP. Of 195 respondents for ‘importance of communication’, 68% marked the two highest categories (important and most important; Table 2). Of 194 respondents, 88% were in the top two categories for ‘importance of patient understanding SUDEP concerns’. Twenty‐four of 195 responses indicated that SUDEP should always be discussed with all patients (12%).

**TABLE 2:** Respondents views on SUDEP communication overall, UK and Norway.

Question	Category	UK (*N* = 197)		Norway (*N* = 112)		*p* value
		*n*	*n* (%)	*n*	*n* (%)	
Importance of SUDEP communication	1 (not important)	195	2 (1%)	112	0 (0%)	**<0.001**
	2		14 (7%)		15 (13%)	
	3		47 (24%)		43 (38%)	
	4		51 (26%)		37 (33%)	
	5 (important)		81 (42%)		17 (15%)	
Importance that patient understands concerns	1 (not important)	194	1 (1%)	110	1 (1%)	**<0.001**
	2		4 (2%)		6 (5%)	
	3		19 (10%)		26 (24%)	
	4		48 (25%)		35 (32%)	
	5 (very important)		122 (63%)		42 (38%)	
Factors influencing not discussing SUDEP^a^	Time availability	195	66 (34%)	110	30 (27%)	0.24
	Complexity		45 (23%)		33 (30%)	0.18
	Low clinical risk		98 (50%)		65 (59%)	0.14
	Distress of patient		85 (44%)		56 (51%)	0.22
	Ability comprehend		75 (38%)		32 (29%)	0.10
	Other reason		22 (11%)		15 (14%)	0.55
	None (always)		24 (12%)		6 (5%)	0.05
Views on FTF and virtual consultations^a^	FTFand virtual same	192	92 (48%)	104	24 (23%)	**<0.001**
	Virtual less likely		43 (22%)		58 (56%)	**<0.001**
	Better face to face		79 (41%)		32 (31%)	0.08
	Better virtually		5 (3%)		1 (1%)	0.67
Patient died of SUDEP	No	193	84 (44%)	111	72 (65%)	**<0.001**
	Yes		109 (56%)		39 (35%)	
Bereavement support available	No	181	78 (43%)	86	74 (86%)	**<0.001**
	Yes		103 (57%)		12 (14%)	
Overall experience of counselling	Negative	192	14 (7%)	109	1 (1%)	0.44
	Neither positive or negative		112 (58%)		70 (64%)	
	Positive		66 (34%)		38 (35%)	

Abbreviations: FTF, face to face; SUDEP, sudden unexpected death in epilepsy.

Bold values indicate p < 0.05 considered as statistically significant.

^a^ Respondents could answer in more than one category. Percentage values may not add up to 100%.

For 195 respondents, the most frequent factor for *not* discussing SUDEP were ‘low clinical risk’ (50%), ‘not distressing the patient’ (44%), the ‘patients’ ability to comprehend’ (38%), ‘clinical complexity’ (23%) and ‘time availability’ (34%; Figure 1).

Almost half of 192 respondents felt that there was no difference between face to face and virtual communication when discussing SUDEP. However, if asked to choose one over the other, face to face was preferred. Twenty‐two per cent of responders were less likely to discuss SUDEP virtually.

Of 181 responses, 57% were aware that bereavement support was available. Where this was known and needed, 65% signposted patients to this support. Of 192 responses, 58% had neither a positive or negative view of SUDEP counselling. However, of the responses who provided a view, more had a positive experience of referring to counselling (34%) than a negative one (7%).

#### Comparison by professionals

The professional groups varied significantly in the proportion of work that was epilepsy specific (*p* < 0.001). Of the 80 responses from nurses, 90% spent 75% or more time on epilepsy‐specific work. Nurses were more likely to discuss SUDEP with all patients than other professions (*p* = 0.008; Table 3).

**TABLE 3:** UK: Background demographics and epilepsy factors by professional role.

Question	Category	Neurologists (*N* = 47)		Psychiatrists (*N* = 14)		Paediatricians (*N* = 42)		Nurses (*N* = 80)		*p* value
		*n*	*n* (%)	*n*	*n* (%)	*n*	*n* (%)	*n*	*n* (%)	
Experience in epilepsy	0–5 years	47	2 (4%)	14	4 (29%)	41	6 (15%)	80	38 (48%)	**<0.001**
	5–10 years		15 (32%)		3 (21%)		8 (20%)		16 (20%)	
	10–15 years		10 (21%)		4 (29%)		10 (24%)		10 (13%)	
	>15 years		20 (43%)		3 (21%)		17 (41%)		16 (20%)	
Percentage work epilepsy specific	<25%	47	17 (36%)	14	9 (64%)	42	2 (5%)	80	3 (4%)	**<0.001**
	25%–50%		11 (23%)		4 (29%)		19 (45%)		4 (5%)	
	50%–75%		12 (26%)		0 (0%)		15 (36%)		1 (1%)	
	>75%		7 (15%)		1 (7%)		6 (14%)		72 (90%)	
How often discuss SUDEP^a^	All patients	46	5 (11%)	13	2 (15%)	42	4 (10%)	80	25 (31%)	**0.008**
	Only new patients		24 (52%)		3 (23%)		24 (57%)		44 (55%)	0.17
	If change in risk		35 (76%)		9 (69%)		33 (79%)		55 (69%)	0.64
	Patients who ask		26 (57%)		1 (8%)		26 (62%)		39 (49%)	**0.006**
	When remember		3 (7%)		1 (8%)		0 (0%)		0 (0%)	**0.02**
	Rarely or never		0 (0%)		0 (0%)		1 (2%)		0 (0%)	0.30
Percentage time discuss SUDEP	<25%	43	42 (98%)	13	11 (85%)	39	38 (97%)	72	61 (85%)	**0.03**
	≥25%		1 (2%)		2 (15%)		1 (3%)		11 (15%)	

Abbreviation: SUDEP, sudden unexpected death in epilepsy.

Bold values indicate p < 0.05 considered as statistically significant.

^a^ Respondents could answer in more than one category. Percentage values may not add up to 100%.

#### SUDEP awareness by professionals

The four professional groups (neurologists/nurses/psychiatrists/paediatricians) varied significantly in their views on the importance of SUDEP communication and importance of patient understanding (*p* < 0.001; Table 4). Neurologists were least likely to indicate that either issue were important.

**TABLE 4:** UK: SUDEP awareness by professional role.

Question	Category	Neurologists (*N* = 47)		Psychiatrists (*N* = 14)		Paediatricians (*N* = 42)		Nurses (*N* = 80)		*p* value
		*n*	*n* (%)	*n*	*n* (%)	*n*	*n* (%)	*n*	*n* (%)	
Importance of SUDEP communication	1 (not important)	47	1 (2%)	13	0 (0%)	42	1 (2%)	80	0 (0%)	**<0.001**
	2		8 (17%)		0 (0%)		4 (10%)		1 (2%)	
	3		13 (28%)		4 (31%)		16 (38%)		9 (11%)	
	4		11 (23%)		2 (15%)		12 (29%)		24 (30%)	
	5 (important)		14 (30%)		7 (54%)		9 (21%)		46 (58%)	
Importance that patient understands concerns	1 (not important)	47	0 (0%)	13	0 (0%)	42	1 (2%)	80	0 (0%)	**<0.001**
	2		2 (4%)		0 (0%)		1 (2%)		1 (1%)	
	3		5 (11%)		0 (0%)		8 (19%)		4 (5%)	
	4		22 (47%)		1 (8%)		11 (26%)		13 (16%)	
	5 (very important)		18 (38%)		12 (92%)		21 (50%)		62 (78%)	
Factors influencing not discussing SUDEP^a^	Time availability	47	20 (43%)	13	0 (0%)	42	13 (31%)	80	27 (38%)	**0.04**
	Complexity		14 (30%)		1 (8%)		5 (12%)		20 (25%)	0.11
	Low clinical risk		29 (62%)		9 (69%)		23 (55%)		31 (39%)	**0.03**
	Distress of patient		23 (49%)		4 (31%)		14 (33%)		38 (48%)	0.29
	Ability comprehend		23 (49%)		1 (8%)		13 (31%)		32 (40%)	**0.04**
	Other reason		3 (6%)		2 (15%)		5 (12%)		12 (15%)	0.53
	None (always)		5 (11%)		2 (15%)		5 (12%)		10 (13%)	0.97
Views on FTF and virtual consultations^a^	FTF and virtual same	46	19 (41%)	13	9 (69%)	41	16 (39%)	80	42 (53%)	0.16
	Virtual less likely		13 (28%)		1 (8%)		14 (34%)		12 (15%)	**0.04**
	Better face to face		21 (46%)		3 (23%)		17 (41%)		33 (41%)	0.54
	Better virtually		0 (0%)		0 (0%)		1 (2%)		4 (5%)	0.48
Patient died of SUDEP	No	47	23 (49%)	13	8 (62%)	42	13 (31%)	79	35 (44%)	0.17
	Yes		24 (51%)		5 (38%)		29 (69%)		44 (56%)	
Bereavement support available	No	44	17 (38%)	13	4 (31%)	41	21 (51%)	71	30 (2%)	0.52
	Yes		27 (61%)		9 (69%)		20 (49%)		41 (58%)	
Signpost to support^b^	No	27	10 (37%)	9	4 (44%)	20	7 (35%)	41	13 (32%)	0.90
	Yes		17 (63%)		5 (56%)		13 (65%)		28 (68%)	
Overall experience of counselling	Negative	47	3 (6%)	13	1 (8%)	41	4 (10%)	78	4 (5%)	0.89
	Neither positive or negative		30 (64%)		8 (62%)		23 (56%)		46 (59%)	
	Positive		14 (30%)		4 (31%)		14 (34%)		28 (36%)	

Abbreviations: FTF, face to face; SUDEP, sudden unexpected death in epilepsy.

Bold values indicate p < 0.05 considered as statistically significant.

^a^ Respondents could answer in more than one category. Percentage values may not add up to 100%.

^b^ For subjects who indicated only that support was available.

The time spent discussing SUDEP was highest for psychiatrists and nurses compared to neurologists or paediatricians (*p* = 0.03). Neurologists were the most likely to indicate ‘time availability’ (*p* = 0.04), ‘low clinical risk’ (*p* = 0.03) and ‘patient/carer ability to comprehend’ (*p* = 0.04) as reasons for ‘not discussing SUDEP’.

The four groups did not vary significantly in the categories of having a patient die of SUDEP, bereavement support, or overall views of SUDEP counselling.

### Norway

Of the 112 responses, 49% were from neurologists (general/epileptologists), 19% were epilepsy nurse specialists (Table 1).

Of 110 responses for epilepsy clinical experience, 17% had less than 5 years, whilst 45% had over 15 years' experience. More than one in three of the 112 responders worked with PWE more than 75% of the time; 29% worked directly in epilepsy care less than 25% of the time.

#### SUDEP discussion

Two of the 112 respondents discussed SUDEP with all patients, with 11% discussing SUDEP with ‘new patients’ (Figure 1). Respondents were more likely to discuss SUDEP if the patient asked (41%) or if there was a change in risk (39%). Twenty‐six per cent ‘rarely or never discussed SUDEP’. Ninety‐one of 102 indicated that SUDEP discussion took up less than 25% of the time available.

#### SUDEP awareness

Of 111 responses, 35% had experienced a SUDEP. Of the 112 responses, 48% indicated that the importance of communication was in the two highest categories, whilst 13% did not feel it was of importance (Table 2). Seventy per cent of 110 responses felt it important for patients to understand SUDEP concerns.

In 110 responses the most common factors for *not* discussing SUDEP were ‘low clinical risk’ (59%), ‘distress of patient’ (51%), ‘multiple complexity’ (30%) and the ‘patients’ ability to comprehend’ (29%; Figure 1).

Twenty‐four of 104 responses stated that face to face and virtual communication were similar (23%). However, if asked to prefer one over the other, face to face was preferred.

Only the minority (12/86, 14%) were aware of any bereavement support being available. Almost two‐thirds of professionals (64%) had no obvious experience of SUDEP counselling.

#### Comparison by professionals

The analyses focused on the difference between the two main job categories, neurologists and nurses (Table 5). The results suggested differences between neurologists and nurses in terms of the amount of work that was epilepsy specific (*p* < 0.001). 54% of the 37 nurses spent 75% or more time on epilepsy, compared to 22% of the 55 neurologists that responded. Neurologists were more likely to discuss SUDEP if there was a ‘change in risk’ (60% vs. 6%, *p* < 0.001), whilst nurses were more likely to discuss with ‘patients who ask’ (54% vs. 33%, *p* = 0.04).

**TABLE 5:** Norway: Background demographics and epilepsy factors by professional role.

Question	Category	Neurologists (*N* = 55)		Nurses (*N* = 37)		*p* value
		*n*	*n* (%)	*n*	*n* (%)	
Experience in epilepsy	0–5 years	54	8 (15%)	36	6 (17%)	0.28
	5–10 years		14 (26%)		6 (17%)	
	10–15 years		11 (20%)		4 (11%)	
	> 15 years		21 (39%)		20 (56%)	
Percentage work epilepsy specific	<25%	55	24 (44%)	37	4 (11%)	**<0.001**
	25%–50%		10 (18%)		7 (19%)	
	50%–75%		9 (16%)		6 (16%)	
	>75%		12 (22%)		20 (54%)	
How often discuss SUDEP^a^	All patients	55	1 (2%)	37	0 (0%)	1.00
	Only new patient		8 (15%)		2 (5%)	0.31
	If change in risk		33 (60%)		6 (16%)	**<0.001**
	Patients who ask		18 (33%)		20 (54%)	**0.04**
	When remember		3 (5%)		2 (5%)	1.00
	Rarely or never		10 (18%)		13 (35%)	0.07
Percentage time discuss SUDEP	<25%	53	44 (83%)	31	30 (97%)	0.08
	≥25%		9 (17%)		1 (3%)	

Abbreviation: SUDEP, sudden unexpected death in epilepsy.

Bold values indicate p < 0.05 considered as statistically significant.

^a^ Respondents could answer in more than one category. Percentage values may not add up to 100%.

#### SUDEP awareness by professionals

The two staff groups did not vary on their views on the importance of SUDEP communication or the importance of patient understanding (Table 6). Neurologists were more likely than nurses to indicate ‘low clinical risk’ as reasons for not discussing SUDEP than nurses (*p* = 0.02). Neurologists were also more likely to indicate ‘time availability’ as a reason for not discussing SUDEP than nurses (approaching significance, *p* = 0.06). There were no differences in any other responses.

**TABLE 6:** Norway: SUDEP awareness by professional role.

Question	Category	Neurologists (*N* = 55)		Nurses (*N* = 37)		*p* value
		*n*	*n* (%)	*n*	*n* (%)	
Importance of SUDEP communication	1 (not important)	55	0 (0%)	37	0 (0%)	0.16
	2		8 (15%)		6 (16%)	
	3		23 (42%)		9 (24%)	
	4		19 (35%)		14 (38%)	
	5 (important)		5 (9%)		8 (22%)	
Importance that patient understands concerns	1 (not important)	55	0 (0%)	36	1 (3%)	0.16
	2		5 (9%)		1 (3%)	
	3		16 (29%)		8 (22%)	
	4		19 (35%)		11 (31%)	
	5 (very important)		15 (27%)		15 (42%)	
Factors influencing not discussing SUDEP^a^	Time availability	55	19 (35%)	36	6 (17%)	0.06
	Complexity		19 (35%)		9 (25%)	0.34
	Low clinical risk		39 (71%)		17 (47%)	**0.02**
	Distress of patient		33 (60%)		17 (47%)	0.23
	Ability comprehend		17 (31%)		7 (19%)	0.23
	Other reason		4 (7%)		6 (17%)	0.16
	None (always)		3 (15%)		2 (6%)	0.98
Views on FTF and virtual consultations^a^	FTF and virtual same	55	8 (15%)	33	10 (30%)	0.08
	Virtual less likely		36 (65%)		16 (48%)	0.12
	Better face to face		16 (29%)		9 (27%)	0.86
	Better virtually		0 (0%)		1 (3%)	0.38
Use of structured tools	No	52	49 (94%)	33	30 (91%)	0.56
	Yes		3 (6%)		3 (9%)	
Patient died of SUDEP	No	55	35 (64%)	36	24 (67%)	0.77
	Yes		20 (36%)		12 (33%)	
Bereavement support available	No	49	45 (92%)	24	18 (75%)	0.05
	Yes		4 (8%)		6 (25%)	
Overall experience of counselling	Negative	55	0 (0%)	35	0 (0%)	0.98
	Neither positive or negative		36 (65%)		23 (66%)	
	Positive		19 (35%)		12 (34%)	

Abbreviations: FTF, face to face; SUDEP, sudden unexpected death in epilepsy.

Bold values indicate p < 0.05 considered as statistically significant.

^a^ Respondents could answer in more than one category. Percentage values may not add up to 100%.

The views on face to face or virtual consultations did not vary significantly between the groups, and nor did the use of structured tools or the experience of having a patient die of SUDEP.

### Comparison: UK and Norway

There were differences in the professional roles of respondents between the UK and Norway (*p* < 0.001; Figure 1, Table S4). Neurologists made 58% of responses from Norway but only around 26% of those from the UK. Conversely, paediatricians made up 23% of UK responses, whilst there were only two responses from paediatricians in Norway. The Norwegian cohort was generally more experienced, with 62% having at least 10 years of experience compared to 49% of the UK group (*p* = 0.01).

The two countries did not vary in terms of the percentage of time that was epilepsy specific or the percentage of time that SUDEP was discussed. Respondents from the UK were more likely to discuss with all patients (*p* < 0.001), only with new patients (*p* < 0.001) and if the risk changes (*p* < 0.001). Those from Norway were more likely to ‘rarely or never discuss SUDEP’ (*p* < 0.001).

### SUDEP awareness comparison between countries

UK participants placed more importance than their Norwegian counterparts on SUDEP communication and patient understanding of SUDEP (*p* < 0.001; Table 2). UK respondents were more likely to consider face to face consultations the same as virtual (*p* < 0.001) and more likely to consider SUDEP counselling virtually (*p* < 0.001). UK respondents were more likely to have had a patient die of SUDEP (*p* < 0.001) and were also more likely to be aware of access to bereavement support services to signpost to (*p* < 0.001).

### Neurologists: comparison between UK and Norway

The two countries’ neurologists did not vary in terms of experience or time spent in epilepsy work (Tables S1 and S5). The UK neurologists were more likely to discuss SUDEP with new patients and with ‘patients who ask’. Norwegian neurologists were more likely to ‘rarely or never discuss SUDEP’ (18% vs. 0%, *p* = 0.002). However, Norwegian neurologists spent more time if discussing SUDEP than those from the UK (*p* = 0.02).

Both countries’ neurologists did not vary in terms of their views on the ‘importance of SUDEP communication’ (Table S1). However, more importance was attributed to ‘patient understanding concerns’ by UK neurologists (*p* = 0.03). There was no difference in the factors of why SUDEP was not discussed.

UK neurologists were more likely not to have a preference in discussing SUDEP virtually or face to face (*p* = 0.002). Over 61% of UK neurologists had awareness of bereavement support available, compared to only 8% of neurologists from Norway (*p* < 0.001).

### Nurses: comparison between UK and Norway

Norwegian nurses were more epilepsy experienced but spent less time on epilepsy than their UK counterparts (*p* < 0.001; Tables S2 and S3). UK nurses were more likely to discuss SUDEP with ‘all patients’ (*p* < 0.001) and ‘only new patients’ (*p* < 0.001), and ‘if there is a change in risk’ (*p* < 0.001). Norway nurses were more likely to ‘rarely or never discuss SUDEP’ (35% vs. 0%, *p* < 0.001).

UK nurses placed more importance on ‘SUDEP communication’ (*p* < 0.001) and ‘patients understating concerns’ (*p* < 0.001). However, UK nurses were less likely to discuss SUDEP if someone did not have the ‘ability to comprehend’ (*p* = 0.03). Norway nurses were less likely to discuss SUDEP on virtual consultations (*p* < 0.001). UK respondents were more likely to indicate no difference between the two consultation methods (*p* = 0.03). UK nurses had more experience of a patient dying of SUDEP, with 56% having experienced this compared to 25% of Norwegian nurses (*p* = 0.03). Bereavement support services were more likely to be recognised to be available by UK nurses (*p* = 0.006).

### Free‐text analysis

Six recurring themes were identified, four of which were present in both countries’ respondents and two predominantly amongst the Norwegian respondents. Table S6 provides example quotes relating to the respective themes. Both country groups’ themes included recognition of the emotional response of patients to SUDEP communication, risk, need for factual representation and concerns of the ambiguity of SUDEP awareness. With respect to emotional response, respondents alluded to the fear discussing SUDEP can evoke in patients and carers.

Responses centred around risk included acknowledgement of risk as well as patient collaboration to facilitate risk reduction. Responses relating to focusing on facts suggest a felt need to maintain objectivity within the context of professional consultations. Respondents alluded to the tension of providing SUDEP awareness whilst some patients and relatives have pre‐existing awareness of it.

Norwegian‐respondent‐specific themes included having a lack of knowledge and/or experience pertaining to discussing SUDEP, as well as not considering SUDEP to be a priority topic.

## DISCUSSION

This was the first implementation of a survey on SUDEP counselling developed from previously published SUDEP survey evidence [11]. The response from 309 (UK 197, Norway 112) clinicans is commensurate with other similar surveys based upon country size and dissemination methods [11].

A key finding is a positive opinion shift on ‘when’ and ‘with whom’ SUDEP counselling should take place. This is particularly evident with the UK data indicating that only 2% ‘rarely or never discuss SUDEP’. The results from Norway are still lower than previous large‐scale surveys (from UK, Europe, North America and Eastern Mediterranean) where non‐discussion ranges from 42% to 68% [25, 26, 27, 28]. There is recognition that geography plays a significant role in SUDEP related matters [29].

UK responders compared to Norway were more likely to convey greater importance to SUDEP communication and to ensuring patients understand SUDEP, largely influenced by nursing. UK nurses were more likely to discuss SUDEP in general than Norway nurses. The role of the nurse in UK epilepsy care differs significantly from Norway's, with nurses taking a more prominent role in SUDEP counselling. In Norway, most nurses consider SUDEP counselling to be a task for the treating physician.

Attitudes of neurologists and nurses in the UK and Norway suggest an overall difference in the cultural approach. In the UK there is an active third sector, clinical and research lobby for SUDEP [2]. Work done by patient‐facing organizations has increased awareness and provided UK clinicians with an established communication tool to facilitate communicating SUDEP [6, 16, 22, 30]. In Norway, there is no active lobbying for increased information about SUDEP, neither amongst physicians, patients nor support groups. There is a common consensus amongst clinicians that information about SUDEP should be given to patients but there is an uncertainty whether all patients should be informed.

The UK responders were more likely to be able to identify bereavement support. Again, awareness and access to bereavement support might have been influenced by charitable advocacy agencies in the UK.

Six themes were identified, four of which were evident in both UK and Norwegian responders. Participants felt that SUDEP discussion could elicit ‘fear’. This concern aligns with previous findings where 36% of PWE and their caregivers reported increased fear following education about SUDEP [31]. However, despite such fears, 95% felt that practitioners should deliver SUDEP education [31].

‘Risk’ was another identified theme. Risk mitigation should address modifiable risk factors as part of an individualized epilepsy care plan [14, 15, 16].

The theme relating to ‘facts’ indicates the need to provide objective information relating to SUDEP, consistent with the National Institute for Health and Care Excellence guidance, which recommends provision of factual material to patients and carers [12]. However, it might also stem from anxiety in communicating multi‐faceted risk in a stratified and patient‐centred manner [14, 15, 16, 20].

The fourth theme common to both countries was ‘awareness’. Some patients/carers may have SUDEP‐related awareness. However, concerns exists in presuming such knowledge as many patients lack this [32]. A further concern would be that SUDEP awareness is not the same as knowing individual risks and impact [20].

Norway‐specific themes involved first a lack of knowledge and experience in communication. This suggests the need for training and possible provision of evidence‐based semi‐structured tools to build confidence in clinicians to discuss a sensitive topic such as SUDEP [16, 20]. UK epilepsy nurses have their own association and competence framework that outlines their professional role clinically and in education [33]. This includes clinical review, epilepsy care plans and risk assessments. A key competence is SUDEP counselling.

The other theme was considering SUDEP a non‐priority topic even though all current best practice suggests that discussion of SUDEP is imperative [31, 32]. The boom in social media and search engines even 10 years ago highlighted that the search online for SUDEP grew significantly [34].

### Limitations

The survey results should be viewed in the context of the responder rate that is low compared to the total number of potential responders, although numerous avenues for dissemination were used. Any survey cannot demonstrate causation, only association. Although this survey was developed from an evidence‐based review of implemented surveys there was no pilot testing performed prior to dissemination that may have helped check reliability based upon respondents' feedback.

## CONCLUSION

### Clinical implications

Clinicians should feel confident to undertake person‐centred holistic discussions, avoiding paternalistic approaches of categorizing patients as low risk, which removes opportunities for patient education, engagement and informed decision making (Table 7). Focus needs to be moved from spot risk assessments to longitudinal understanding of disease change and associated health and social behaviour. Health system change is necessary to provide more time and support for sensitive conversations to occur repeatedly from first appointment onwards, especially given that risks can quickly become fatal between appointments [34, 35].

**TABLE 7:** A practical clinical approach towards reducing SUDEP risk [12, 13, 14, 15, 30, 40, 41, 42, 43, 44].

Potentially modifiable risk factors	Clinical management
Seizure frequency (generalized tonic–clonic seizures)	Maximize treatment with anti‐seizure medication whilst considering therapeutic balance between efficacy and adverse effects. This includes risk of anti‐cholinergic effects that accumulate with overall pharmacological load and more evident as people get older Aim for seizure freedom. However, many people are treatment resistant. Therefore, aiming to reduce seizure burden, particularly reduce tonic–clonic seizures. Less than three GTC seizures per year associated with reduced risk of seizures
Nocturnal seizures	Night‐time surveillance, e.g. audio/visual monitor. Other measures and technologies may support risk management plan; however, have limited evidence base to date, with low sensitivity and specificity. Avoid prone sleeping position
Concordance with medication	Work collaboratively with person with epilepsy to maintain good compliance with treatment regime. Explore rationale for low compliance such as adverse effects (including specifically adverse effects on sexual function). Routine: once daily dosing with longer acting medicines, formulation and taste, interaction with other prescribed medicines
Regular specialist reviews/epilepsy care plans	Minimum annual review of epilepsy care plans and individualized risk assessments. Minimum annual review with epilepsy specialist, particularly if other comorbidities, e.g. neurodevelopmental disorders
Communication	Clinicians and services to provide integrated care with communication between services based upon a personalized epilepsy care plan that is readily accessible. Multidisciplinary team involvement to support wider comorbidities (e.g. psychiatric, alcohol/substance misuse) may reduce risk

*Note*: (1) SUDEP to be discussed at the earliest appropriate opportunity following diagnosis. (2) SUDEP risk to be reviewed regularly as part of the epilepsy management. (3) Consider validated SUDEP risk assessment tools, and background screening (cardiac, genetic).

Abbreviations: GTC, generalized tonic–clonic; SUDEP, sudden unexpected death in epilepsy.

### Policy implications

Epilepsy remains outside of UK health and policy priorities, despite the World Health Organization Intersectoral Global Action Plan placing it as an urgent priority. With 76% of epilepsy deaths potentially preventable, it is vital to alleviate clinical barriers to discussing SUDEP and wider epilepsy mortality risks [35]. The study, particularly the Norwegian responses, show significant potential for developing communication practices for the benefit and safety of all PWE from diagnosis and beyond. Our study suggests that, even in high income countries such as UK and Norway with high levels of education and literacy, there is a need to develop SUDEP communication practices for the benefit and safety of all patients.

### Research implications

Further research into impactfully and positively communicating SUDEP risks and empowering patients' risk self‐management is urgently needed. There might be a role to consider evidence‐based self‐empowerment using digital tools [37, 38, 39].

## AUTHOR CONTRIBUTIONS

**Lance Watkins:** Investigation; writing – original draft; methodology; validation. **Oliver Henning:** Conceptualization; investigation; writing – original draft; methodology; validation. **Paul Bassett:** Formal analysis; data curation; validation; methodology; writing – review and editing. **Samantha Ashby:** Conceptualization; writing – review and editing; methodology; visualization. **Samuel Tromans:** Formal analysis; data curation; visualization; validation; writing – review and editing. **Rohit Shankar:** Conceptualization; investigation; validation; methodology; writing – review and editing; project administration; supervision; resources.

## FUNDING INFORMATION

This research did not receive any specific grant from funding agencies in the public, commercial or not‐for‐profit sectors.

## CONFLICT OF INTEREST STATEMENT

OH has received honoraria from LivaNova, Eisai, UCB, Novartis, Desitin and Jazz for work outside the submitted manuscript/project. SA is CEO of the national charity SUDEP Action. RS has received institutional and research support from LivaNova, UCB, Eisai, Veriton Pharma, Bial, Angelini, UnEEG and Jazz/GW pharma outside the submitted work. He holds grants from NIHR AI, SBRI and other funding bodies all outside this work. No other author has any declared conflict of interest related to this paper.

## ETHICS STATEMENT

The authors confirm that they have read the journal's position on issues involved in ethical publication and affirm that this report is consistent with those guidelines.

## Supporting information


Data S1.

Data S2.

Data S3.

Table S1.
Table S2.
Table S3.
Table S4.
Table S5.
Table S6.

## Data Availability

The data that support the findings of this study are available from the corresponding author upon reasonable request.

## References

[1] Nashef L , So EL , Ryvlin P , Tomson T . Unifying the definitions of sudden unexpected death in epilepsy. Epilepsia. 2012;53(2):227‐233. doi:10.1111/j.1528-1167.2011.03358.x 22191982

[2] Walczak TS , Leppik IE , D'Amelio M , et al. Incidence and risk factors in sudden unexpected death in epilepsy: a prospective cohort study. Neurology. 2001;56(4):519‐525. doi:10.1212/wnl.56.4.519 11222798

[3] Saetre E , Abdelnoor M . Incidence rate of sudden death in epilepsy: a systematic review and meta‐analysis. Epilepsy Behav. 2018;86:193‐199. doi:10.1016/j.yebeh.2018.06.037 30017838

[4] Ryvlin P , Nashef L , Lhatoo SD , et al. Incidence and mechanisms of cardiorespiratory arrests in epilepsy monitoring units (MORTEMUS): a retrospective study. Lancet Neurol. 2013;12(10):966‐977. doi:10.1016/S1474-4422(13)70214-X 24012372

[5] Friedman D , Kannan K , Faustin A , et al. Cardiac arrhythmia and neuroexcitability gene variants in resected brain tissue from patients with sudden unexpected death in epilepsy (SUDEP). NPJ Genom Med. 2018;3:9. doi:10.1038/s41525-018-0048-5 29619247 PMC5869741

[6] Sveinsson O , Andersson T , Mattsson P , Carlsson S , Tomson T . Clinical risk factors in SUDEP: a nationwide population‐based case–control study. Neurology. 2020;94(4):e419‐e429. doi:10.1212/WNL.0000000000008741 31831600 PMC7079690

[7] Shankar R , Donner EJ , McLean B , Nashef L , Tomson T . Sudden unexpected death in epilepsy (SUDEP): what every neurologist should know. Epileptic Disord. 2017;19(1):1‐9. doi:10.1684/epd.2017.0891 28218059

[8] Shankar R , Henley W , Boland C , et al. Decreasing the risk of sudden unexpected death in epilepsy: structured communication of risk factors for premature mortality in PWE. Eur J Neurol. 2018;25(9):1121‐1127. doi:10.1111/ene.13651 29611888

[9] Shankar R , Newman C , Gales A , et al. Has the time come to stratify and score SUDEP risk to inform PWE of their changes in safety? Front Neurol. 2018;9:281. doi:10.3389/fneur.2018.00281 29755403 PMC5934492

[10] Mclean B , Shankar R , Hanna J , Jory C , Newman C . Sudden unexpected death in epilepsy: measures to reduce risk. Pract Neurol. 2017;17(1):13‐20. doi:10.1136/practneurol-2016-001392 27903766

[11] Watkins LV , Ashby S , Hanna J , Henley W , Laugharne R , Shankar R . An evidence‐based approach to provide essential and desirable components to develop surveys on sudden unexpected death in epilepsy (SUDEP) for doctors: a focused review. Seizure. 2023;106:14‐21. doi:10.1016/j.seizure.2023.01.007 36706666

[12] National Institute for Health and Care Excellence (NICE) . Clinical Guidelines (NG217) Epilepsies in children, young people and adults. 2022. 35700280

[13] Harden C , Tomson T , Gloss D , et al. Practice guideline summary: sudden unexpected death in epilepsy incidence rates and risk factors: report of the guideline development, dissemination, and implementation Subcommittee of the American Academy of Neurology and the American Epilepsy Society. Epilepsy Curr. 2017;17(3):180‐187. doi:10.5698/1535-7511.17.3.180 28684957 PMC5486432

[14] Watkins L , Shankar R , Sander JW . Identifying and mitigating sudden unexpected death in epilepsy (SUDEP) risk factors. Expert Rev Neurother. 2018;18(4):265‐274. doi:10.1080/14737175.2018.1439738 29425076

[15] Watkins L , Shankar R . Reducing the risk of sudden unexpected death in epilepsy (SUDEP). Curr Treat Options Neurol. 2018;20(10):40. doi:10.1007/s11940-018-0527-0 30136125

[16] Shankar R , Ashby S , McLean B , Newman C . Bridging the gap of risk communication and management using the SUDEP and seizure safety checklist. Epilepsy Behav. 2020;103(Pt B):106419. doi:10.1016/j.yebeh.2019.07.020 31648927

[17] Aurlien D , Larsen JP , Gjerstad L , Taubøll E . Increased risk of sudden unexpected death in epilepsy in females using lamotrigine: a nested, case–control study. Epilepsia. 2012;53:258‐266. doi:10.1111/j.1528-1167.2011.03334.x 22126371

[18] Henning O , Nakken KO , Lossius MI . PWE and their relatives want more information about risks of injuries and premature death. Epilepsy Behav. 2018;82:6‐10. doi:10.1016/j.yebeh.2018.02.023 29574300

[19] Public Health England: The Burden of Disease in England compared with 22 peer countries A report for NHS England. https://assets.publishing.service.gov.uk/media/5e1735f2ed915d3b0b00c7cc/GBD_NHS_England_report.pdf

[20] Smart C , Page G , Shankar R , Newman C . Keep safe: the when, why and how of epilepsy risk communication. Seizure. 2020;78:136‐149. doi:10.1016/j.seizure.2020.01.013 32122784

[21] Shankar R , Newman C , Hanna J , et al. Keeping patients with epilepsy safe: a surmountable challenge? BMJ Open Quality. 2015;4:u208167.w3252. doi:10.1136/bmjquality.u208167.w3252 PMC4645845 26734336

[22] Brown S , Shankar R , Cox D , McLean BM , Jory C . Clinical governance: risk assessment in SUDEP. Clin Govern Int J. 2013;18:325‐331. doi:10.1108/CGIJ-12-2012-0045

[23] Chafe R . The value of qualitative description in health services and policy research. Healthc Policy. 2017;12(3):12‐18. 28277201 PMC5344360

[24] Doyle L , McCabe C , Keogh B , Brady A , McCann M . An overview of the qualitative descriptive design within nursing research. J Res Nurs. 2020;25(5):443‐455. doi:10.1177/1744987119880234 34394658 PMC7932381

[25] Friedman D , Donner EJ , Stephens D , Wright C , Devinsky O . Sudden unexpected death in epilepsy: knowledge and experience among U.S. and Canadian neurologists. Epilepsy Behav. 2014;35:13‐18. doi:10.1016/j.yebeh.2014.03.022 24785429 PMC4176608

[26] Strzelczyk A , Zschebek G , Bauer S , et al. Predictors of and attitudes toward counseling about SUDEP and other epilepsy risk factors among Austrian, German, and Swiss neurologists and neuropediatricians. Epilepsia. 2016;57(4):612‐620. 26899504 10.1111/epi.13337

[27] Saleh DA , Kassie S , Hassan A , Alsaadi T . Sudden unexpected death in epilepsy: a pilot study on neurologists' knowledge and experience in the eastern Mediterranean region. Seizure. 2022;94:57‐65. doi:10.1016/j.seizure.2021.11.011 34864253

[28] Asadi‐Pooya AA , Trinka E , Brigo F , et al. Counseling about sudden unexpected death in epilepsy (SUDEP): a global survey of neurologists' opinions. Epilepsy Behav. 2022;128:108570. doi:10.1016/j.yebeh.2022.108570 35093831

[29] Kinney MO , McCluskey G , Friedman D , Walker MC , Sander JW , Shankar R . Investigative practice into sudden death in epilepsy: a global survey. Acta Neurol Scand. 2019;139(5):476‐482. doi:10.1111/ane.13080 30776083

[30] Shankar R , Cox D , Jalihal V , Brown S , Hanna J , McLean B . Sudden unexpected death in epilepsy (SUDEP): development of a safety checklist. Seizure. 2013;22(10):812‐817. doi:10.1016/j.seizure.2013.07.014 23962523

[31] Long L , Cotterman‐Hart S , Shelby J . To reveal or conceal? Adult patient perspectives on SUDEP disclosure. Epilepsy Behav. 2018;86:79‐84. doi:10.1016/j.yebeh.2018.06.026 30001909

[32] Xu Z , Ayyappan S , Seneviratne U . Sudden unexpected death in epilepsy (SUDEP): what do patients think? Epilepsy Behav. 2015;42:29‐34. doi:10.1016/j.yebeh.2014.11.007 25499158

[33] Tittensor P , Tittensor S , Chisanga E , Bagary M , Jory C , Shankar R . UK framework for basic epilepsy training and oromucosal midazolam administration. Epilepsy Behav. 2021;122:108180. doi:10.1016/j.yebeh.2021.108180 34252835

[34] Brigo F , Igwe SC , Ausserer H , et al. Why do people Google epilepsy? An infodemiological study of online behavior for epilepsy‐related search terms. Epilepsy Behav. 2014;31:67‐70. doi:10.1016/j.yebeh.2013.11.020 24361764

[35] Mbizvo GK , Schnier C , Simpson CR , Chin RFM , Duncan SE . A national study of epilepsy‐related deaths in Scotland: trends, mechanisms, and avoidable deaths. Epilepsia. 2021;62:2667‐2684. doi:10.1111/epi.17065 34537957

[36] Shankar R , Jalihal V , Walker M , et al. A community study in Cornwall UK of sudden unexpected death in epilepsy (SUDEP) in a 9‐year population sample. Seizure. 2014;23(5):382‐385. 24630808 10.1016/j.seizure.2014.02.005

[37] Shankar R , Newman C , McLean B , Anderson T , Hanna J . Can technology help reduce risk of harm in patients with epilepsy? Br J Gen Pract. 2015;65(638):448‐449. doi:10.3399/bjgp15X686413 26324472 PMC4540375

[38] Newman C , Ashby S , McLean B , Shankar R . Improving epilepsy management with EpSMon: a templar to highlight the multifaceted challenges of incorporating digital technologies into routine clinical practice. Epilepsy Behav. 2020;103(Pt B):106514. doi:10.1016/j.yebeh.2019.106514 31526645

[39] Zhou SM , McLean B , Roberts E , et al. Analysing patient‐generated data to understand behaviours and characteristics of women with epilepsy of childbearing years: a prospective cohort study. Seizure. 2023;108:24‐32. doi:10.1016/j.seizure.2023.04.008 37060628

[40] Hesdorffer DC , Tomson T , Benn E , et al. Combined analysis of risk factors for SUDEP. Epilepsia. 2011;52(6):1150‐1159. 21671925 10.1111/j.1528-1167.2010.02952.x

[41] Tomson T , Surges R , Delamont R , Haywood S , Hesdorffer DC . Who to target in sudden unexpected death in epilepsy prevention and how? Risk factors, biomarkers, and intervention study designs. Epilepsia. 2016;57(S1):4‐16. 26749012 10.1111/epi.13234

[42] Langan Y , Nashef L , Sander JW . Case–control study of SUDEP. Neurology. 2005;64(7):1131‐1133. 15824334 10.1212/01.WNL.0000156352.61328.CB

[43] Ryvlin P , Cucherat M , Rheims S . Risk of sudden unexpected death in epilepsy in patients given adjunctive antiepileptic treatment for refractory seizures: a meta‐analysis of placebo‐controlled randomised trials. Lancet Neurol. 2011;10(11):961‐968. 21937278 10.1016/S1474-4422(11)70193-4

[44] Lennard S , Newman R , McLean B , et al. Improving nocturnal event monitoring in people with intellectual disability in community using an artificial intelligence camera. Epilepsy Behav Rep. 2023;22:100603. 37152695 10.1016/j.ebr.2023.100603 PMC10160340

