# Supplementary material for: Epilepsy professionals' views on sudden unexpected death in epilepsy counselling: A tale of two countries
Source: Eur J Neurol. 2024 Jun 4;31(9):e16375. doi: 10.1111/ene.16375 (PMC11295158; doi:10.1111/ene.16375)
Supplement: Supplementary file 2 — Data S2. [file ENE-31-e16375-s001.pdf]

Professional attitudes to SUDEP communication

Dear colleague,
the recent NICE 2022 guidelines again reiterate what has been laid out as best practice i.e. the need to communicate and discuss Sudden Death in Epilepsy (SUDEP) with people with epilepsy.

This is a brief survey of professionals who work primarily with children and adults with epilepsy to help understand what the practice, challenges and possible barriers are for SUDEP communication at present. Many professionals may counsel about SUDEP as part of counselling about epilepsy being a life-threatening condition – however this survey focuses specifically on SUDEP communication, rather than wider epilepsy mortality risks.

The survey is supported by SUDEP Action. It would allow us to gain insight to the unique problems faced by professionals working in the field (example - training, time & resource issues etc.) and take action to support clinicians suitably and raise the issue in the right spaces. The collated results and analysis of the survey will be fed back in national and international conferences, newsletters and an academic publication.

All questions are optional and majority multiple choice. We envisage the survey to take a minimum of 10 -12 minutes. The survey is designed to keep your replies anonymous. We would presume informed consent if you submit the survey.

We appreciate this is a busy time for you and you could be getting various other requests for information. We do hope this survey output can be representative of your needs and views to make a direct impact on service delivery. We are very grateful for your consideration of this.

1. Principal clinical job role (if currently training please identify the closest professional role you are in)

Mark only one oval.

- Neurologist - general
- Neurologist - epileptologist
- Epilepsy specialist nurse
- Nurse - other
- Psychiatrist -Neuropsychiatrist
- Psychiatrist - Intellectual disabilities/developmental disabilities
- Psychiatrist - other
- General Paediatrician
- Paediatric neurologist/epileptologist
- Other: ______________________

2. Years of experience in epilepsy professional role

Mark only one oval.

- 0-5 years
- 5-10 years
- 10-15 years
- >15 years

3. What proportion of your work is epilepsy specific?

Mark only one oval.

- <25%
- 25-50%
- 50-75%
- >75%

4. How often do you discuss SUDEP with your patients who have epilepsy? (please select all applicable)

Check all that apply.

- [ ] All patients and/or their carers/family - no exceptions in every appointment
- [ ] Only new patients and/or their carers/families
- [ ] Patients and/or their families in whom I perceive a change in risk in clinical appointments
- [ ] Patients and/or their family members/carers who ask me of SUDEP
- [ ] Only when I remember to
- [ ] rarely or never

5. If possible provide a short explanation for your choice above

____

6. When you discuss SUDEP with a patient and/or carer/family on an average what % of your allocated appointment time would you spend on it?

Mark only one oval.

- ( ) <25%
- ( ) 25-50%
- ( ) >50%
- ( ) Other: ______________________________

7. How important in the management of an individual's epilepsy do you believe clinician SUDEP communication is?

Mark only one oval.

not important at all

1 ◯

2 ◯

3 ◯

4 ◯

5 ◯

one of the most important issues

8. How important from your perspective is it for the patient and/or their family/carer to have understood about SUDEP concerns?

Mark only one oval.

Not important at all

1 ◯
2 ◯
3 ◯
4 ◯
5 ◯

Very important

9. What factors influence your likelihood to not have a discussion of SUDEP (please select all applicable)

Check all that apply.

- [ ] Time availability
- [ ] multiple complexity present
- [ ] Clinical risk perceived low
- [ ] Patient likely to become distressed
- [ ] Patient ability to comprehend - example have a learning disability
- [ ] None of the above (as discussion with all patients all times)
- [ ] Other: ____________________

10. With regard to face to face versus virtual consultations which of these statements represent your views (please select all the applies)

Check all that apply.

- [ ] I communicate SUDEP issues the same way irrespective of it being virtual or face to face consultations
- [ ] I am less likely to discuss SUDEP if virtual as compared to face to face
- [ ] I communicate SUDEP issues virtually or face to face but feel I can do it better face to face
- [ ] I communicate SUDEP issues virtually or face to face but feel I do it better virtually

11. In your opinion what are the common risk factors for SUDEP? Please provide up to 5 factors you consider relevant from your practice.

____

12. Do you use any structured or semi-structured tools, information leaflets, websites or other patient facing resources to discuss/communicate SUDEP risk - if so what?

____

13. Have any of your patients died of SUDEP?

Mark only one oval.

- ( ) Yes
- ( ) No

14. When you hear of a patient's death, do you signpost the bereaved family to specialist epilepsy bereavement support?

Mark only one oval.

- ( ) Yes
- ( ) No
- ( ) Such a service is not available

15. If "yes" is there any particular service or agency?

____

16. How do you find your overall experience of SUDEP counselling for people with epilepsy

Mark only one oval.

- ( ) Positive
- ( ) Negative
- ( ) neither positive or negative

17. If possible please explain your reasons for your answer above

____

18. Thank you very much for your time. If you would like to have a copy of the results or discuss any aspects of this survey further please contact rohit.shankar@plymouth.ac.uk

____

This content is neither created nor endorsed by Google.

Google Forms
