# Supplementary material for: Epilepsy professionals' views on sudden unexpected death in epilepsy counselling: A tale of two countries
Source: Eur J Neurol. 2024 Jun 4;31(9):e16375. doi: 10.1111/ene.16375 (PMC11295158; doi:10.1111/ene.16375)
Supplement: Supplementary file 3 — Data S3. [file ENE-31-e16375-s004.pdf]

Region:	Saksbehandler:	Telefon:	Vår dato:	Vår referanse:
REK sør-øst C	Johanne Holmen	22855260	24.03.2023	607647

Oliver Johannes Henning

Fremleggingsvurdering: Å kommunisere SUDEP – relevans og utfordringer
Søknadsnummer: 607647
Forskningsansvarlig institusjon: Oslo universitetssykehus HF

Prosjektet vurderes som ikke fremleggingspliktig

Søkers beskrivelse

Epilepsi er assosiert med risiko for tidlig sykelighet og dødelighet. En hovedårsak til epilepsirelatert dødelighet er Sudden Unexpected Death in Epilepsy (SUDEP).
Et viktig aspekt av fokus for å redusere SUDEP har vært å kommunisere om SUDEP og de potensielle risikofaktorer til pasienter og/eller deres familier. Det er imidlertid lite litteratur om hva som diskuteres og når det diskuteres av klinikere. Det finnes også lite informasjon om hvordan klinikere finner prosessen med å levere SUDEP-relatert informasjon eller om de bruker spesifikke verktøy og ressurser.
Dette er en kort undersøkelse av fagpersoner for å forstå hva praksis, utfordringer og mulige barrierer er for SUDEP-kommunikasjon i dag.
Lenke til nettbasert spørreskjema vil bli sendt til både leger og sykepleiere via Norsk epilepsiforening, nasjonalt epilepsisykepleiernettverk og kollegaer som jobber i felten.

Vi viser til innsendt fremleggingsvurderingsskjema datert 23.03.2023. Henvendelsen ble behandlet av sekretariatet for Regional komité for medisinsk og helsefaglig forskningsetikk (REK) på fullmakt.

REKs vurdering
En hovedårsak til epilepsirelatert dødelighet er Sudden Unexpected Death in Epilepsy (SUDEP). Formålet med prosjektet, slik det fremgår av framleggingsvurderingen, er å kartlegge praksis, utfordringer og mulige barrierer for SUDEP-kommunikasjon blant helsepersonell. Leger og sykepleiere vil få en lenke til nettbasert spørreskjema. Det innhentes ingen pasientinformasjon eller personlig informasjon. Svarene fra helsepersonellet som deltar i undersøkelsen er vurdert som anonyme. Spørsmålene fremstår som en kartlegging av helsepersonellets erfaring og bruk av SUDEP-veiledning.

Konklusjon
Etter REKs vurdering faller prosjektet, slik det er beskrevet, utenfor virkeområdet til helseforskningsloven.
Helseforskningsloven gjelder for medisinsk og helsefaglig forskning, i loven definert som forskning på mennesker, humant biologisk materiale og helseopplysninger, som har som formål å frambringe ny kunnskap om helse og sykdom, jf. helseforskningsloven §§ 2 og 4a. Formålet er avgjørende, ikke om forskningen utføres av helsepersonell eller på pasienter/sårbare grupper eller benytter helseopplysninger.

Prosjekter som faller utenfor helseforskningslovens virkeområde kan gjennomføres uten godkjenning av REK. Det er institusjonens ansvar på å sørge for at prosjektet gjennomføres på en forsvarlig måte med hensyn til for eksempel regler for taushetsplikt og personvern.

Vi gjør oppmerksom på at vurderingen og konklusjonen er å anse som veiledende jf. forvaltningsloven § 11. Dersom dere likevel ønsker å søke REK vil søknaden bli behandlet i komitémøte, og det vil bli fattet et enkeltvedtak etter forvaltningsloven.

Med vennlig hilsen

Jacob C. Hølen
Sekretariatsleder
REK sør-øst C

Johanne Holmen
Seniorrådgiver

Kopi til:
Oslo universitetssykehus HF
