# Supplementary material for: Epilepsy professionals' views on sudden unexpected death in epilepsy counselling: A tale of two countries
Source: Eur J Neurol. 2024 Jun 4;31(9):e16375. doi: 10.1111/ene.16375 (PMC11295158; doi:10.1111/ene.16375)
Supplement: Supplementary file 4 — Table S1. Table S2. Table S3. Table S4. Table S5. Table S6. [file ENE-31-e16375-s002.docx]

Supplementary Table 1. Comparison of SUDEP Awareness factors between UK and Norway – Neurologists only

| Question | Category | UK [N=47] | | Norway [N=55] | | P-value |
| --- | --- | --- | --- | --- | --- | --- |
|  |  | n | n (%) | n | n (%) |  |
|  |  |  |  |  |  |  |
| Importance of | 1 (not important) | 47 | 1 (2%) | 55 | 0 (0%) | 0.22 |
| SUDEP | 2 |  | 8 (17%) |  | 8 (15%) |  |
| communication | 3 |  | 13 (28%) |  | 23 (42%) |  |
|  | 4 |  | 11 (23%) |  | 19 (35%) |  |
|  | 5 (important) |  | 14 (30%) |  | 5 (9%) |  |
|  |  |  |  |  |  |  |
| Importance that | 1 (not important) | 47 | 0 (0%) | 55 | 0 (0%) | **0.03** |
| patient | 2 |  | 2 (4%) |  | 5 (9%) |  |
| understands | 3 |  | 5 (11%) |  | 16 (29%) |  |
| concerns | 4 |  | 22 (47%) |  | 19 (35%) |  |
|  | 5 (very important) |  | 18 (38%) |  | 15 (27%) |  |
|  |  |  |  |  |  |  |
| Factors | Time availability | 47 | 20 (43%) | 55 | 19 (35%) | 0.41 |
| influencing not | Complexity |  | 14 (30%) |  | 19 (35%) | 0.61 |
| discussing | Low clinical risk |  | 29 (62%) |  | 39 (71%) | 0.33 |
| SUDEP ^(*)^ | Distress of patient |  | 23 (49%) |  | 33 (60%) | 0.26 |
|  | Ability comprehend |  | 23 (49%) |  | 17 (31%) | 0.06 |
|  | Other reason |  | 3 (6%) |  | 4 (7%) | 0.86 |
|  | None (always) |  | 5 (11%) |  | 3 (5%) | 0.33 |
|  |  |  |  |  |  |  |
| Views on FTF | FTF & virtual same | 46 | 19 (41%) | 55 | 8 (15%) | **0.002** |
| & virtual | Virtual less likely |  | 13 (28%) |  | 36 (65%) | **<0.001** |
| consultations ^(*)^ | Better face to face |  | 21 (46%) |  | 16 (29%) | 0.09 |
|  | Better virtually |  | 0 (0%) |  | 0 (0%) | 1.00 |
|  |  |  |  |  |  |  |
| Patient died of | No | 47 | 23 (49%) | 55 | 35 (64%) | 0.14 |
| SUDEP | Yes |  | 24 (51%) |  | 20 (36%) |  |
|  |  |  |  |  |  |  |
| Bereavement | No | 44 | 17 (39%) | 49 | 45 92%) | **<0.001** |
| support avail. | Yes |  | 27 (61%) |  | 4 (8%) |  |
|  |  |  |  |  |  |  |
| Overall | Negative | 55 | 3 (6%) | 55 | 0 (0%) | 0.35 |
| experience of | Neither pos. or neg. |  | 30 (64%) |  | 36 (65%) |  |
| counselling | Positive |  | 14 (30%) |  | 19 (35%) |  |
|  |  |  |  |  |  |  |

(*) Respondents could answer in more than one category. Percentage values may not add up to 100%

Supplementary Table 2: Comparison of demographics and background epilepsy factors between UK and Norway – Nurses only

| Question | Category | UK [N=80] | | Norway [N=37] | | P-value |
| --- | --- | --- | --- | --- | --- | --- |
|  |  | n | n (%) | n | n (%) |  |
|  |  |  |  |  |  |  |
| Experience in | 0 – 5 years | 80 | 38 (48%) | 36 | 6 (17%) | **<0.001** |
| epilepsy | 5 – 10 years |  | 16 (20%) |  | 6 (17%) |  |
|  | 10 – 15 years |  | 10 (13%) |  | 11 (11%) |  |
|  | > 15 years |  | 16 (20%) |  | 56 (56%) |  |
|  |  |  |  |  |  |  |
| % work epilepsy | < 25% | 80 | 3 (4%) | 37 | 4 (11%) | **<0.001** |
| specific | 25% – 50% |  | 4 (5%) |  | 7 (19%) |  |
|  | 50% – 75% |  | 1 (1%) |  | 6 (16%) |  |
|  | > 75% |  | 72 (90%) |  | 20 (54%) |  |
|  |  |  |  |  |  |  |
| How often | All patients | 80 | 25 (31%) | 37 | 0 (0%) | **<0.001** |
| discuss | Only new patient |  | 44 (55%) |  | 2 (5%) | **<0.001** |
| SUDEP ^(*)^ | If change in risk |  | 55 (69%) |  | 6 (16%) | **<0.001** |
|  | Patients who ask |  | 39 (49%) |  | 20 (54%) | 0.59 |
|  | When remember |  | 0 (0%) |  | 2 (5%) | 0.10 |
|  | Rarely or never |  | 0 (0%) |  | 13 (35%) | **<0.001** |
|  |  |  |  |  |  |  |
| % time | < 25% | 72 | 61 (85%) | 31 | 91 (97%) | 0.10 |
| discuss SUDEP | ≥ 25% |  | 11 (15%) |  | 1 (3%) |  |
|  |  |  |  |  |  |  |

(*) Respondents could answer in more than one category. Percentage values may not add up to 100%

Supplementary Table 3. Comparison of SUDEP Awareness factors between UK and Norway – Nurses only

| Question | Category | UK [N=80] | | Norway [N=37] | | P-value |
| --- | --- | --- | --- | --- | --- | --- |
|  |  | n | n (%) | n | n (%) |  |
|  |  |  |  |  |  |  |
| Importance of | 1 (not important) | 80 | 0 (0%) | 37 | 0 (0%) | **<0.001** |
| SUDEP | 2 |  | 1 (1%) |  | 6 (16%) |  |
| communication | 3 |  | 9 (11%) |  | 9 (24%) |  |
|  | 4 |  | 24 (30%) |  | 14 (38%) |  |
|  | 5 (important) |  | 46 (58%) |  | 8 (22%) |  |
|  |  |  |  |  |  |  |
| Importance that | 1 (not important) | 80 | 0 (0%) | 36 | 1 (3%) | **<0.001** |
| patient | 2 |  | 1 (1%) |  | 1 (3%) |  |
| understands | 3 |  | 4 (16%) |  | 8 (22%) |  |
| concerns | 4 |  | 13 (16%) |  | 11 (31%) |  |
|  | 5 (very important) |  | 62 (78%) |  | 15 (42%) |  |
|  |  |  |  |  |  |  |
| Factors | Time availability | 80 | 27 (34%) | 36 | 6 (17%) | 0.06 |
| influencing not | Complexity |  | 20 (25%) |  | 9 (25%) | 1.00 |
| discussing | Low clinical risk |  | 31 (39%) |  | 17 (47%) | 0.39 |
| SUDEP ^(*)^ | Distress of patient |  | 38 (48%) |  | 17 (47%) | 0.98 |
|  | Ability comprehend |  | 32 (40%) |  | 7 (19%) | **0.03** |
|  | Other reason |  | 10 (13%) |  | 2 (6%) | 0.26 |
|  | None (always) |  | 12 (15%) |  | 6 (17%) | 0.82 |
|  |  |  |  |  |  |  |
| Views on FTF | FTF & virtual same | 80 | 42 (53%) | 33 | 10 (30%) | **0.03** |
| & virtual | Virtual less likely |  | 12 (15%) |  | 16 (48%) | **<0.001** |
| consultations ^(*)^ | Better face to face |  | 33 (41%) |  | 9 (27%) | 0.16 |
|  | Better virtually |  | 4 (5%) |  | 1 (3%) | 1.00 |
|  |  |  |  |  |  |  |
| Patient died of | No | 55 | 84 (44%) | 36 | 72 (65%) | **0.03** |
| SUDEP | Yes |  | 109 (56%) |  | 39 (35%) |  |
|  |  |  |  |  |  |  |
| Bereavement | No | 71 | 30 (42%) | 24 | 18 (75%) | **0.006** |
| support avail. | Yes |  | 41 (58%) |  | 6 (25%) |  |
|  |  |  |  |  |  |  |
| Overall | Negative | 78 | 4 (5%) | 35 | 0 (0%) | 0.86 |
| experience of | Neither pos. or neg. |  | 46 (59%) |  | 23 (66%) |  |
| counselling | Positive |  | 28 (36%) |  | 12 (34%) |  |
|  |  |  |  |  |  |  |

(*) Respondents could answer in more than one category. Percentage values may not add up to 100%

## Supplementary Table 4: Comparison of demographic and background epilepsy factors between UK and Norway – All staff

| Question | Category | UK [N=197] | | Norway [N=112] | | P-value |
| --- | --- | --- | --- | --- | --- | --- |
|  |  | n | n (%) | n | n (%) |  |
|  |  |  |  |  |  |  |
| Job category | Neurologists | 183 | 47 (26%) | 95 | 55 (58%) | **<0.001** |
|  | Psychiatrists |  | 7 (7%) |  | 1 (1%) |  |
|  | Paediatricians |  | 42 (23%) |  | 2 (2%) |  |
|  | Nurses |  | 80 (44%) |  | 37 (39%) |  |
|  |  |  |  |  |  |  |
| Experience in | 0 – 5 years | 195 | 54 (28%) | 36 | 19 (17%) | **0.01** |
| epilepsy | 5 – 10 years |  | 44 (23%) |  | 23 (21%) |  |
|  | 10 – 15 years |  | 36 (18%) |  | 19 (17%) |  |
|  | > 15 years |  | 61 (31%) |  | 49 (45%) |  |
|  |  |  |  |  |  |  |
| % work epilepsy | < 25% | 196 | 37 (19%) | 112 | 33 (29%) | 0.05 |
| specific | 25% – 50% |  | 39 (20%) |  | 21 (19%) |  |
|  | 50% – 75% |  | 32 (16%) |  | 17 (15%) |  |
|  | > 75% |  | 88 (45%) |  | 41 (37%) |  |
|  |  |  |  |  |  |  |
| How often | All patients | 193 | 39 (20%) | 112 | 2 (2%) | **<0.001** |
| discuss | Only new patient |  | 100 (52%) |  | 12 (11%) | **<0.001** |
| SUDEP ^(*)^ | If change in risk |  | 140 (73%) |  | 44 (39%) | **<0.001** |
|  | Patients who ask |  | 98 (51%) |  | 46 (41%) | 0.10 |
|  | When remember |  | 5 (3%) |  | 7 (6%) | 0.11 |
|  | Rarely or never |  | 3 (2%) |  | 29 (26%) | **<0.001** |
|  |  |  |  |  |  |  |
| % time | < 25% | 178 | 162 (91%) | 102 | 91 (89%) | 0.62 |
| discuss SUDEP | ≥ 25% |  | 16 (9%) |  | 11 (11%) |  |
|  |  |  |  |  |  |  |

(*) Respondents could answer in more than one category. Percentage values may not add up to 100%

Supplementary Table 5: Comparison of demographics and background epilepsy factors between UK and Norway – Neurologists only

| Question | Category | UK [N=47] | | Norway [N=55] | | P-value |
| --- | --- | --- | --- | --- | --- | --- |
|  |  | n | n (%) | n | n (%) |  |
|  |  |  |  |  |  |  |
| Experience in | 0 – 5 years | 47 | 2 (4%) | 54 | 8 (15%) | 0.44 |
| epilepsy | 5 – 10 years |  | 15 (32%) |  | 14 (26%) |  |
|  | 10 – 15 years |  | 10 (21%) |  | 11 (20%) |  |
|  | > 15 years |  | 20 (43%) |  | 21 (39%) |  |
|  |  |  |  |  |  |  |
| % work epilepsy | < 25% | 47 | 17 (36%) | 55 | 24 (44%) | 0.80 |
| specific | 25% – 50% |  | 11 (23%) |  | 10 (18%) |  |
|  | 50% – 75% |  | 12 (26%) |  | 9 (16%) |  |
|  | > 75% |  | 7 (15%) |  | 12 (22%) |  |
|  |  |  |  |  |  |  |
| How often | All patients | 46 | 5 (11%) | 55 | 1 (2%) | 0.09 |
| discuss | Only new patient |  | 24 (52%) |  | 8 (15%) | **<0.001** |
| SUDEP ^(*)^ | If change in risk |  | 35 (76%) |  | 33 (60%) | 0.09 |
|  | Patients who ask |  | 26 (56%) |  | 18 (33%) | **0.02** |
|  | When remember |  | 3 (7%) |  | 3 (5%) | 0.82 |
|  | Rarely or never |  | 0 (0%) |  | 10 (18%) | **0.002** |
|  |  |  |  |  |  |  |
| % time | < 25% | 43 | 42 (98%) | 53 | 44 (83%) | **0.02** |
| discuss SUDEP | ≥ 25% |  | 1 (2%) |  | 9 (17%) |  |
|  |  |  |  |  |  |  |

(*) Respondents could answer in more than one category. Percentage values may not add up to 100%

Supplementary Table 6. Recurring themes among free-text response question items

| **Theme** | **Example Quotes** | |
| --- | --- | --- |
|  | **UK respondents** | **Norway respondents** |
| Emotional response | “Although sometimes it enlists terror in people, they feel much better being aware and knowing of the risks, than not knowing.” | “I have parenting education here in the children's department and have therefore spoken to the former boss and professional nurses about this topic, where I have been advised not to talk about it because it often creates more fear in parents than it helps them.” |
| Risk | “I find that people generally have an awareness of SUDEP and i build on on their knowledge helping the person to explore their understanding of their risks and how we can work together in the aim of reducing the risk.” | “Most people appreciate knowing about the elevated risk of sudden unexpected death.” |
| Focussing on facts | “I believe it needs to be discussed with in a matter of fact manner, unless i feel the patient is at higher risk then i would be more forceful in highlighting the dangers.” | “Everyone has heard of SUDEP, it is our job to explain it in a good and factual way without trivializing or dramatizing.” |
| Awareness | “People are aware of SUDEP, although they do not enjoy discussing it, I say that I have to discuss this and also provide a leaflet to take home” | “When you mention it to patients or relatives, it often turns out that they have heard of it before.” |
| Lack of knowledge and/or experience | N/A | “I have little to no experience talking about SUDEP, this is what the patient's neurologist talks to the patient about.” |
| Not a priority topic | N/A | “I have very rarely been in a situation where this has been relevant. A few times patients have brought up the topic as a concern. But SUDEP does not come up as a frequent concern.” |
